# Analysis of clinical characteristics of centrally mediated abdominal pain syndrome, exploration of diagnostic markers and its relationship with the efficacy of duloxetine treatment

**DOI:** 10.1097/MD.0000000000032134

**Published:** 2022-12-02

**Authors:** Yuming Tang, Jiani Song, Ying Zhu, Hefeng Chen, Weiyan Yao, Duowu Zou

**Affiliations:** a Department of Gastroenterology, Shanghai Jiao Tong University Medical School Affiliated Ruijin Hospital, Shanghai, China; b Department of Pharmacy, Shanghai Jiao Tong University Medical School Affiliated Ruijin Hospital, Shanghai, China.

**Keywords:** centrally mediated abdominal pain syndrome (CAPS), duloxetine, micro RNA, prospective cohort study

## Abstract

Centrally mediated abdominal pain syndrome (CAPS) is characterized by severe abdominal pain. Diagnosis of CAPS is still an exclusionary diagnosis, there remain no effective diagnostic biomarkers so far. Duloxetine is the major pharmacotherapy of CAPS, while some CAPS patients do not respond to duloxetine treatment. However, there is a lack of molecular markers to predict the efficacy of duloxetine. In our pilot study, we have found differential expression profiles of serum miRNAs between CAPS patients and healthy controls. Our study aims to explore the clinical characteristics, specific miRNAs in serum as diagnostic biomarkers of CAPS and predictive biomarkers of the efficacy of duloxetine.

**Methods/design:**

In this prospective cohort study, we plan to enroll 430 participants including 215 CAPS patients and 215 healthy controls. The CAPS group takes duloxetine 30 mg per day as an initial dose. Patients will have 24-week medication period and follow up at week 0, 4, 12, 24 and 36. Blood samples will be obtained from patients at every visits and health controls at the initial visit and a series of questionnaires will be completed by the participants. The primary end points are: The differential expression of miRNAs between CAPS groups and healthy control groups at baseline. The changes in abdominal pain scores before and after duloxetine treatment in patients with CAPS and their relationship with the changes in miRNAs. The secondary end point is the changes in scores of depression, anxiety, sleep quality and quality of life before and after duloxetine treatment in patients with CAPS and their relationship with changes in miRNAs.

**Discussion:**

Findings of study will provide the reliable basis for diagnosis and the predictor of duloxetine efficacy of CAPS. Importantly, findings grant patients a chance to benefit from treatment.

## 1. Introduction

According to Rome IV criteria, centrally mediated abdominal pain syndrome (CAPS) is characterized by continuous, nearly continuous, or frequently recurrent abdominal pain that is often severe and only rarely related to gut function. CAPS is associated with loss of function across several life domains, including work, intimacy, social/leisure and must be present for at least 6 months before diagnosis.^[1]^

The prevalence of CAPS ranges from 0.5% to 2.1% which is lower than other functional gastrointestinal diseases, such as irritable bowel syndrome (IBS) and functional dyspepsia.^[2]^ However, it is worth note that CAPS has a relatively serious impact on daily life among functional gastrointestinal diseases. Chronic and recurrent pain was the main reason why people visits to gastroenterology outpatient clinic repeatedly.^[3]^ Most of them take numerous unnecessary examinations, receive negative results. Heavy socioeconomic costs include utilization of medical resources and unemployment due to illness.^[4]^ However, the diagnosis of CAPS remains difficult. At present, based on ROME IV criteria, diagnosis of CAPS mainly depends on symptoms, duration and which is an exclusionary diagnosis. It is essential to find a specific and sensitive biomarkers to make a precise and rapid CAPS diagnosis.

Previous studies demonstrated that CAPS resulted from central sensitization with disinhibition of pain signals rather than increased peripheral afferent excitability.^[5]^ Psychological factors will influence human pain perception and down-regulate pain inhibition. Published literacy demonstrated that antidepressants and psychological therapy were potential therapy approaches. Duloxetine is a new SNRIs, mainly inhibits the serotonin and norepinephrine reuptake simultaneously.^[6]^ Current evidence demonstrated that Duloxetine can improve CAPS patients’ frequency of abdominal pain and anxiety.^[7]^ A cohort study of 103 patients found that 53% of patients (n = 10/19, *P* = .007) responded using 1 centrally acting neuromodulator, and effectiveness of duloxetine in CAPS group was 71%.^[8]^ Despite duloxetine seemed to be the most potent single agent neuromodulator, a clutch of patients get unsatisfactory effect. Unfortunately, there is currently a lack of means to predict therapeutic efficacy of duloxetine.

In the field of pain, current study has focused on 2 mechanisms, DNA methylation and miRNA expression.^[9]^ Micro RNAs are a class of a small noncoding RNAs that capable of silencing and post-transcriptional regulation of gene expression. Researchers have found grater variability of miRNA expression between IBS patients and healthy control, such as miR-199a-5p, miR-199b-5p, miR-29a3p, miR-219a-5p, miR-338-3p.^[10]^ Helena Kyunghee Kim et al found that miR-16-5p, hsa-miR-146a-5p and hsa-miR-21-5p were a potential predictive biomarkers of duloxetine response in patients with depression.^[11]^ In our pilot study, we also found differential expression profiles of serum miRNAs between CAPS patients and healthy controls. We therefore hypothesized that these miRNAs could be a potential biomarkers to diagnosis CAPS and predict efficacy of duloxetine.

To date, there is little information available regarding miRNA expression profile in serum of CAPS and predictor of duloxetine efficacy. Thus our study aims to explore the specific miRNAs in serum to solve this problem.

We present the protocol in accordance with the SPIRIT reporting checklist.

## 2. Methods and analysis

### 2.1. Study design

This study is a prospective cohort study conducted in China (ChiCTR2200064371). Our study aims to explore serum miRNAs as a diagnostic biomarker and a predictor of efficacy of duloxetine for patients with CAPS.

Blood samples will be taken from participants who visited our hospital. A total of 215 CAPS patients and 215 healthy controls will be enrolled from the inpatient and outpatient departments of gastroenterology and the digestive endoscopy center respectively.

### 2.2. Study hypothesis

We hypothesized that: there is differential expression of miRNAs between CAPS group and healthy control group; duloxetine would improve abdominal pain; and miRNAs may be associated with the prognosis of patients with CAPS after duloxetine treatment.

### 2.3. Study purpose

1. To evaluate the differential expression of miRNAs in patients with CAPS and healthy controls, to provide new biomarkers for diagnosis of CAPS.
2. To explore the association between the changes of miRNAs and the efficacy of duloxetine treatment at week 4, 12, 24 and 36 in patients with CAPS.

### 2.4. Studying settings and recruitment

This study is now conducted at Ruijin Hospital from October 1, 2022 to December 31, 2023. We decide to recruit CAPS patients from the inpatient and outpatient of departments of gastroenterology. Meanwhile, the healthy controls will be recruited from the individuals who visit the digestive endoscopy center for routine checkups of gastroscopy and colonoscopy. All participants who experienced physicians will be screened for enrollment eligibility. Diseases will also be diagnosed by experienced gastroenterologists. To enhance participants compliance, they will make appointment for next visit when outpatient clinical visits and will be reminded via telephone or message.

### 2.5. Eligibility criteria

#### 2.5.1. Inclusion criteria.

All CAPS patients must meet all of the following criteria: age at 18 to 85; meet the Rome IV diagnostic criteria; a signed and dated informed consent form; and commit to comply with study process and align with implementing the whole study process

All healthy controls must meet all of the following criteria: age at 18 to 85; no abdominal pain and other gastrointestinal symptoms; no organic lesions at gastroenterological endoscopy in latest 6 months; a signed and dated informed consent form; and commit to comply with study process and align with implementing the whole study process.

#### 2.5.2. Exclusion criteria.

Participants who meet either of the following criteria will be excluded: participants complicated with organic lesions; participants with contraindications to duloxetine; patients who are taking other antidepressant; participants with a history of intellectual disability, impaired consciousness, mental disorders, seizures; pregnant and lactating female; patients meet the diagnostic criteria for any psychiatric disorder and Statistical Manual of Mental Disorders criteria of depression or anxiety, diagnosed depression or anxiety; with Hamilton Anxiety Scale (HAMA) scores more than 14, Hamilton Depression Scale scores more than 18; with high blood pressure  > 140/90 mm Hg; with a history of recent abdominal surgery; with a history of ulcerative colitis, Crohn disease, celiac disease and a family history of colorectal cancer; with a history of angle-closure glaucoma, uncontrolled thyroid disease and tumor; patients who are unwilling or unable to comply with the study protocol or cannot to cooperate with follow-up; and participants who are considered unsuitable by the investigator to participate in this study.

### 2.6. Intervention

#### 2.6.1. Drugs.

The property of duloxetine (produced by LILLY DEL CARIBE, Inc) is white or off-white enteric-coated granules. 30 mg capsule: white opaque capsule body and blue capsule cap, 30 mg printed on the capsule; 60 mg capsule: white opaque capsule body and blue capsule cap, 60 mg printed on the capsule. Package: aluminum blister pack, 7 capsules per box, 14 capsules per box, 28 capsules per box.

Withdrawal of medications must be taper to complete withdrawal, and monitor the withdrawal response. If intolerance, return to the previously dose, and then reduction with lesser dose. Duloxetine should be swallowed, not chewed or crushed.

All drugs use was recorded by Hospital information system. All scales and scores were recorded and saved by computer, which were used to evaluate intervention.

#### 2.6.2. Intervention flow.

**Figure 1.**
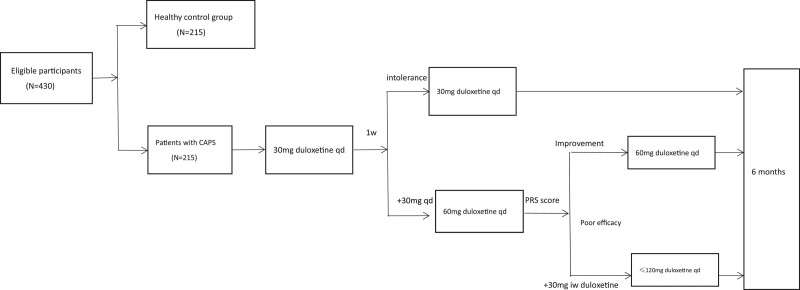
Intervention flow. CAPS = centrally mediated abdominal pain syndrome, iw = once every 2 weeks, PRS = pain relief scale, qd = once per day, w = week.

The intervention flow is illustrated in Figure 1.

### 2.7. Study procedure and timeline

**Figure 2.**
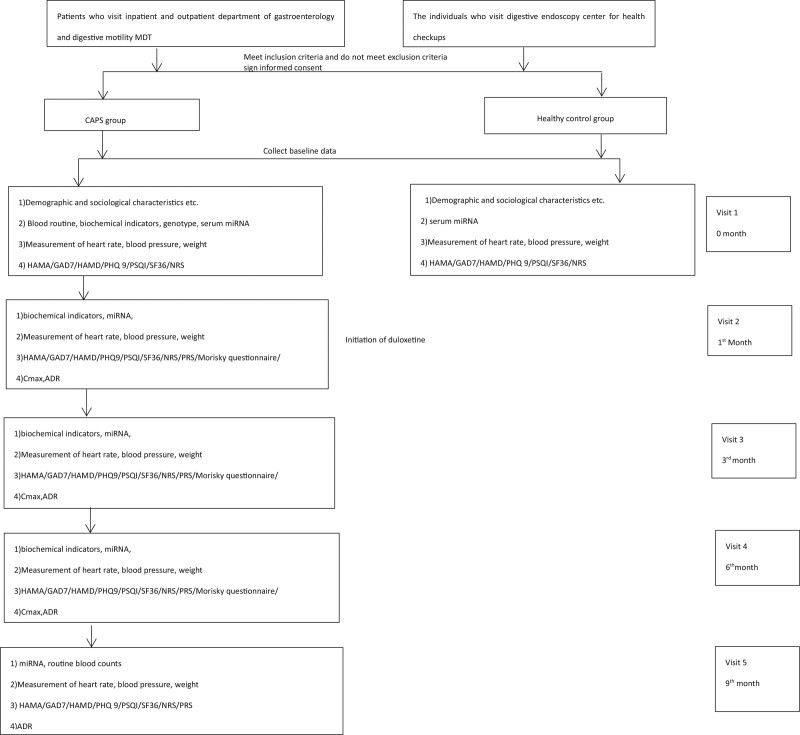
Study procedure. ADR = adverse drug reaction, BP = blood pressure, Cmax = maximum serum concentration, GAD7 = Generalized Anxiety Disorder7, HAMA = Hamilton anxiety scale, HAMD = Hamilton depression scale, HR = heart rate, NRS = numerical rating scale, PHQ9 = patient health questionaires 9, PRS = pain relief scale, PSQI = Pittsburgh sleep quality index, QoL = quality of life, SF36 = short form 36.

The study procedure and timeline is illustrated as Figure 2 and Table 1. Heart rate, blood pressure, weight will be recorded at every visits. At the initial visit (visit1), patients will be diagnosed with CAPS by an experienced gastroenterologist. Patients and healthy controls will be screened by a experienced gastroenterologist. All of them will sign an informed consent form. Demographic and baseline data will be collected. The subjects will be asked to complete a series of questionnaires in order to get baseline scores. Baseline scores on the Numerical Rating Scale, the HAMA, Generalized Anxiety Disorder7, Hamilton Depression Scale, Patient Health Questionaires 9, Short Form 36, Pittsburgh sleep quality index will be obtained. Blood routine, biochemical indicators and genotype will also be carried out in the laboratory of Ruijin Hospital. On the ground of experimental method of Real-Time Fluorescence Quantitative PCR, the serum miRNAs will be sequenced between patients with CAPS and healthy control group.

**Table 1:** Study timeline.

Visits	Screening period	Treatment period		Therapy termination	Follow-up period
	1	2	3	4	5 (Follow-up 1)
Treatment period (mo)	0	1	3	6	9
Informed consent form	x				
Demography and baseline	x				
Inclusion/exclusion	x				
NRS	x	x	x	x	x
HAMA/HAMD/PSQI/GAD7/PHQ9/SF36	x	x	x	x	x
PRS		x	x	x	x
QoL	x	x	x	x	x
Morisky questionnaire		x	x	x	
HR	x	x	x	x	x
BP	x	x	x	x	x
Weight	x	x	x	x	x
miRNA	x	x	x	x	x
Routine blood counts	x				x
Liver and renal function	x	x	x	x	
Cmax		x	x	x	
ADR		x	x	x	x
Genotype	x				

ADR = adverse drug reaction, BP = blood pressure, Cmax = maximum serum concentration, GAD7 = generalized anxiety disorder7, HAMA = Hamilton anxiety scale, HAMD = Hamilton depression scale, HR = heart rate, NRS = numerical rating scale, PHQ9 = patient health questionaires 9, PRS = pain relief scale, PSQI = Pittsburgh sleep quality index, QoL = quality of life, SF36 = short form 36.

After the initial visit, every patient starts to take duloxetine under the instruction of gastroenterologist. The initial dose is 30 mg qd. After 1 week, if tolerated, increase dose to 60 mg qd, or 30 mg qd continued. In the duration of therapy, altering dose of duloxetine based on pain relief scale (PRS) score. We suggest adding 30 mg every 2 weeks, however, the total dose should not exceed 120 mg qd.

During the treatment period, patients need to have 3 outpatient visits as follow-up including 1 month (visit 2), 3 month (visit 3) and 6 months (visit 4) after the treatmet, respectively. Patients will be required to complete the same questionnaires as at the initial visit and 2 additional questionnaires, including PRS and Morisky questionnaire. Biochemical indicators, Maximum serum concentration of duloxetine, blood miRNA will be tested in the laboratory of Ruijin Hospital. Adverse drug reaction will also be recorded.

After 24-week treatment period, there is a further 12-week outpatient post-treatment follow-up period. At the terminal visit (visit 5), patients will be required to complete all the rating scales except Morisky questionnaire. Adverse drug reaction will also be recorded. Micro RNA, genotype and blood routine will be tested again.

### 2.8. Criteria for discontinuing the intervention

Sever adverse reactions; Any other disease which affects the efficacy of duloxetine, and determination of Adverse events; Patient is deemed unsuitable for continuing to take duloxetine by the researchers; Subjects withdraw informed consent form; Patients are reluctant to continue their treatment because of poor efficacy; and Lost to follow up.

The reasons for discontinuation and withdrawal would be recorded on the case report form. Subjects who signed informed consent but did not accept the intervention will be replaced. Subjects who signed informed consent, accept the intervention and subsequently withdraw will or may be not replaced.

### 2.9. Lost to follow-up

Participant stops scheduled study follow-up; participant fails to complete study-prescribed procedures; and loss the connection with participant.

### 2.10. Outcomes measures

#### 2.10.1. Primary outcome.

1. The differential expression of miRNAs between CAPS groups and healthy control groups at baseline.
2. The changes in abdominal pain scores before and after duloxetine treatment in patients with CAPS and their relationship with changes in miRNA.

#### 2.10.2. Secondary outcome.

The changes in depression, anxiety, sleep quality and quality of life scores before and after duloxetine treatment in patients with CAPS and their relationship with changes in miRNA.

### 2.11. Data collection

#### 2.11.1. Questionnaire.

HAMA Generalized Anxiety Disorder7/Hamilton Depression Scale Patient Health Questionaires 9: reflect subjects’ psychological status (anxiety/depression)

Pittsburgh sleep quality index: Pittsburgh sleep quality index. To evaluate sleep quality score

Short Form 36: Short form 36. To evaluate quality of life score

Numerical Rating Scale: Numerical Rating Scale. To evaluate change in severity of abdominal pain before and after duloxetine treatment

PRS: To evaluate whether duloxetine dose adjustment was required.

Morisky questionnaire: To evaluate medication adherence.

#### 2.11.2. Physical measurements.

Standardized measurements of heart rate and weight will be performed and blood pressure will be measured by electronic sphygomanometer in order to minimize the potential data deviation and gain realistic data.

#### 2.11.3. Laboratory measurements.

In Clinical Laboratory of Shanghai Ruijin Hospital, blood routine and biochemical indicators will be tested. In laboratory of Gastroenterology, based on the experimental method of Real-Time Fluorescence Quantitative PCR, the miRNA will be sequenced.

### 2.12. Statistical analysis

#### 2.12.1. Sample size calculation.

Taking the bilateral test α = 0.05 and 1-β (power of test) = 0.95, the total sample size of the patients group is 172 cases. According to the 20% shedding or elimination possibility, the sample size of patients group is 215 cases. According to the allocation 1:1, the healthy control group is 215 cases.

#### 2.12.2. Data analysis.

Statistical Package for the Social Sciences for Mac book air, version 26 will be used for data analysis. For descriptive statistics, we use proportions for categorical variables, mean ± SD for normal continuous data, median for non-normally continuous data. K-S tests will be used for the normality test. Descriptive statistic is used to describe the demographic characteristics and laboratory test results. Whole data of primary and secondary outcomes are repeated measure and continuous data. X2 test is used for comparison at different time points and intergroup differences in term of baseline data. If the difference is detected statistically significant, post hoc multiple pairwise comparison will be performed. The association of blood biomarkers, plasma concentration, genotype and clinical efficacy, medication adherence will be assessed by Spearman correlation analysis. *P* < 0.05 is considered statistically significant.

We use intention to treat and per protocol to deal with missing values, outlier values, lost follow-up and non-adherence respectively.

## 3. Discussion

**Figure 3.**
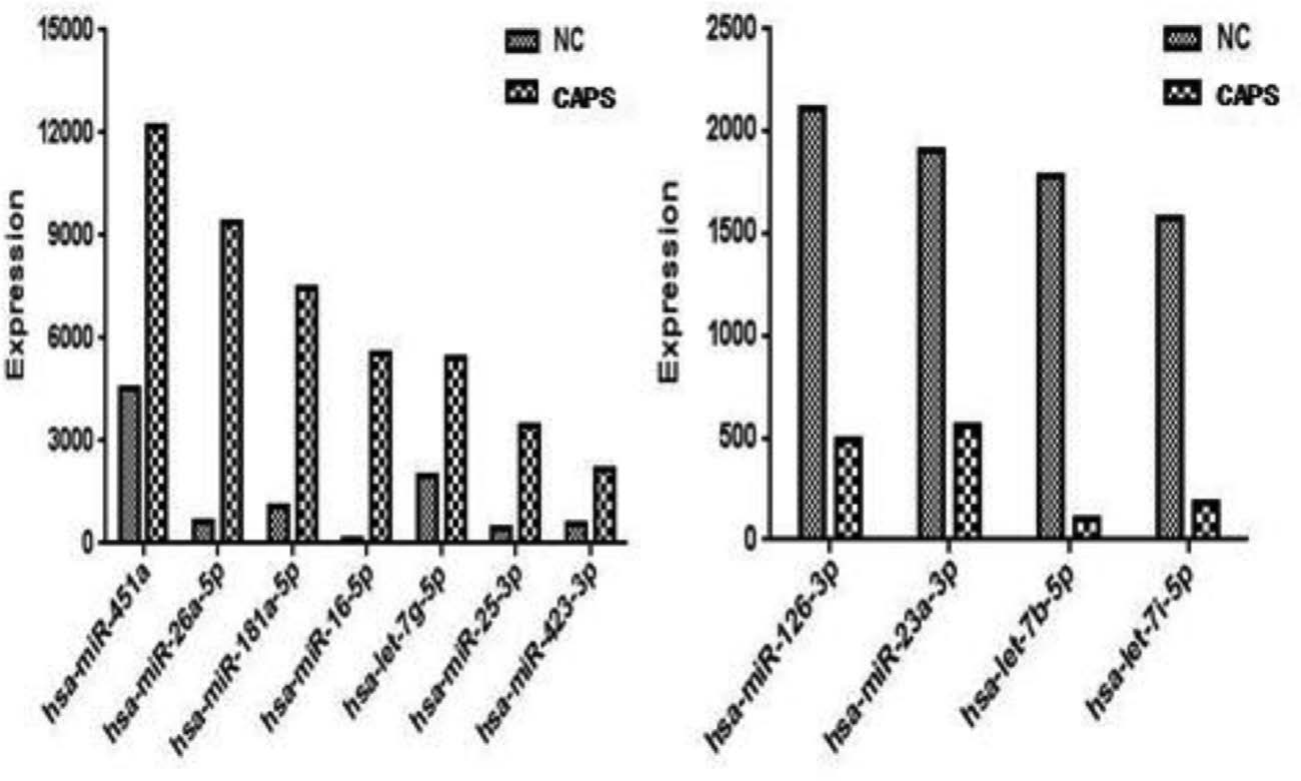
Pilot study result. hsa-miR-415a, hsa-miR-26a-5p, hsa-miR-181a-5p, hsa-miR16-5p, hsa-let-7g-5p, hsa-miR-25-3p, hsa-miR-423-3p were up-regulated and hsa-miR-126-3P, hsa-miR-23a-3p, hsa-let-7b-5p, hsa-let-7i-5p were down-regulated in CAPS group compared with healthy control group. CAPS = centrally mediated abdominal pain syndrome.

CAPS is characterized by chronic pain, which is a major cause of disability in the world.^[12]^ However, accurate diagnosis and treatment of CAPS are still difficult. Therefore, it is warranted to explore the specific biomarkers for CAPS to help the diagnosis and treatment. miRNAs regulate gene expression, significantly extracellular miRNAs are abnormally expressed in serum during the process of disorders.^[13]^ Although studies on miRNA relationship with CAPS were insufficient, the studies on IBS which is another functional gastrointestinal disorders characterized by recurrent abdominal pain, are more in-depth. It has already been show that miR-29 family (29a 29b 29c)was significantly correlated with pathogenesis of IBS by modifying intestinal permeability in IBS patients,^[14]^ furthermore, miRNA-29a reduced HTR7 expression, which due to hypersensitivity in IBS because HTR7 weaken visceral hyperalgesia.^[15]^ Interestingly, in our pilot study, we also found 7 miRNAs, namely hsa-miR-415a, hsa-miR-26a-5p, hsa-miR-181a-5p, hsa-miR16-5p, hsa-let-7g-5p, hsa-miR-25-3p, hsa-miR-423-3p were up-regulated and 4 miRNAs, namely hsa-miR-126-3P, hsa-miR-23a-3p, hsa-let-7b-5p, hsa-let-7i-5p were downregulated in CAPS groups compared with healthy control groups (Fig. 3). Therefore, we can presume that the expression of particular miRNA may become a potential diagnostic biomarkers in the future.

Duloxetine has pain modulating effects which beyond its psychiatric indications, which has been used widely for the functional gastrointestinal disorders.^[16]^ Juan Pablo Lopez et al found that differential expression of miR-146a- 5p, miR-146b-5p, miR-425-3p and miR-24-3p after dolextine treatment for patients with depression in a large, randomized placebo-controlled trial.^[17]^ Accordingly, we believe that miRNA may emerge as a potential predictor of efficacy of duloxetine treatment for patients with CAPS.

Our study has some advantages. Firstly, our study is a prospective study on CAPS. Secondly, our study provides a comprehensive assessment of clinical characteristics, psychological status, quality of life, sleep quality and biomarkers for CAPS. It is worth mentioning that this study may be the first perspective cohort study to explore miRNAs in serum as a diagnostic biomarker and a predictor of efficacy of duloxetine treatment for CAPS.

In a word, this study is helpful to provide the reliable basis for diagnosis and the predictor of duloxetine efficacy of CAPS. Our results will shed significant new light on the development of CAPS.

## Author contributions

Zou Duowu & Yao Weiyan conceived and instructed the study. Tang Yuming designed the study and contributed to the implementation of the trial. Song Jiani contributed to the enrollment, intervention, follow-up of the participants and blood analysis. Zhu Ying contributed to the enrollment, intervention and follow-up of the participants. Chen Hefeng monitored the use and side effects of duloxetine in this study.
